# Comparison of dynamic visual acuity after implantation of toric bifocal or trifocal intraocular lens in age-related cataract patients: a randomized controlled trial

**DOI:** 10.3389/fnins.2023.1287626

**Published:** 2023-12-21

**Authors:** Yuanting Li, Xiaodan Li, Xiaodan Jiang, Yuexin Wang, Tingyi Wu, Huaqin Xia, Xuemin Li

**Affiliations:** ^1^Department of Ophthalmology, Peking University Third Hospital, Beijing, China; ^2^Beijing Key Laboratory of Restoration of Damaged Ocular Nerves, Beijing, China; ^3^Department of Sports Medicine, Peking University Third Hospital, Institute of Sports Medicine of Peking University, Beijing, China; ^4^Beijing Key Laboratory of Sports Injuries, Beijing, China; ^5^Engineering Research Center of Sports Trauma Treatment Technology and Devices, Ministry of Education, Beijing, China

**Keywords:** toric trifocal IOL, toric bifocal IOL, static visual acuity, dynamic visual acuity, age-related cataract

## Abstract

**Purpose:**

To investigate the dynamic visual acuity (DVA) after implantation of toric bifocal or trifocal intraocular lens in age-related cataract patients.

**Methods:**

This was a prospective randomized controlled trial. Of one hundred and twenty-four patients enrolled and randomized to receive unilateral phacoemulsification and toric trifocal (939 M/MP, Carl Zeiss Meditec AG, Jena, Germany) or toric bifocal (909 M, Carl Zeiss Meditec AG, Jena, Germany) intraocular lenses (IOL) implantation, ninety-nine patients completed the follow-up and were included in final analysis. Postoperatively, uncorrected and corrected distance (UDVA and CDVA), intermediate (UIVA and DCIVA) and near (UNVA and DCNVA) static visual acuity, manifest refraction and uncorrected and corrected distance DVA (UDDVA and CDDVA) at 20, 40 and 80 degrees per second (dps) were evaluated at one week, one month and three months.

**Results:**

Three months postoperatively, the UDVA were 0.13 ± 0.11 and 0.14 ± 0.13 in the toric trifocal and bifocal IOL group, respectively. Significant better UIVA (trifocal, 0.17 ± 0.13 vs. bifocal, 0.23 ± 0.13, *p* = 0.037) and DCIVA (trifocal, 0.16 ± 0.11 vs. bifocal, 0.20 ± 0.12, *p* = 0.048) were observed in patients implanting toric trifocal than bifocal IOL at three months postoperatively. Patients implanted with toric bifocal IOL obtained better CDDVA at 80 dps (0.5607 ± 0.2032) than the trifocal group (0.6573 ± 0.2450, *p* = 0.039) at three months. Postoperative UDDVA and CDDVA at 20, 40 and 80 dps were significantly associated with age (*p* < 0.05, respectively) and postoperative static visual acuity (*p* < 0.05, respectively).

**Conclusion:**

Toric trifocal IOL provides better static intermediate visual acuity, and toric bifocal IOL implantation provides better distance dynamic visual acuity at high speed.

## 1 Introduction

Cataract is the leading cause of blindness globally (Lee and Afshari, 2017). As the surgical technique and intraocular lens (IOL) design improve, cataract surgery is far more than preventing blindness. It has evolved into a procedure to improve quality of life by providing a full range of vision and spectacle independence. There are several types of IOLs designed to provide a usable full range of vision (Rampat and Gatinel, 2021; Schallhorn et al., 2021). Multifocal IOL implantation could provide a high spectacle independent rate (de Silva et al., 2016; Modi et al., 2021; Schallhorn et al., 2021). Trifocal and bifocal IOLs are designed to provide three and two focal points, respectively. Previous studies have demonstrated that patients implanted with trifocal IOLs may achieve better intermediate visual acuity (VA), and comparable distance and near VA than bifocal IOLs (de Silva et al., 2016; Shen et al., 2017; Zhang et al., 2021). Pre-existing corneal and residual astigmatism are important factors limiting the application of multifocal IOLs (Woodward et al., 2009; de Vries et al., 2011). The toric multifocal IOL provides a new chance for these patients with good predictability (Gangwani et al., 2014).

Traditionally, static visual acuity, refraction and biometry were primarily used to assess visual function and evaluate the accuracy of intraocular lens implantation efficiency and accuracy after cataract surgery (Hoffer and Savini, 2021; Ouchi, 2022). Since moving objects compose the majority of visual signals in daily life, and sports and driving are increasingly required for postoperative patients, dynamic visual acuity (DVA) is now gradually assessed in cataract patients (Ao et al., 2014; Ren et al., 2020). DVA is the ability to identify details of visual objects with relative movement between observers and visual targets (Wu et al., 2021). Our previous studies have revealed a rehabilitation of DVA after cataract surgery (Ao et al., 2014) and diffractive trifocal IOL implantation provides better dynamic visual acuity than monofocal IOL in age-related cataract patients (Ren et al., 2020). However, the influence of the different types of toric multifocal IOLs in DVA has not been investigated. The present study aims to compare the DVA following implantation of a toric trifocal or bifocal IOL in age-related cataract patients, and investigate potential factors associated with postoperative DVA.

## 2 Methods

### 2.1 Subjects

The present research was a prospective, randomized, double-masked, controlled trial to assess the DVA following monocular implantation of toric trifocal or bifocal IOL in age-related cataract patients. The study was performed following the tenets of the Declaration of Helsinki. The protocol of the study was approved by the ethics committee of Peking University Third Hospital (Approval No. D2021003), and informed consent was obtained from all subjects. The trial was registered at ClinicalTrials.gov (ChiCTR2100046567).

Age-related cataract patients planning to undergo phacoemulsification and IOL implantation were enrolled when they met the following inclusion criteria: (1) age over 18, (2) volunteering to participate in the clinical study and sign informed consent; (3) regular corneal astigmatism ≥0.75D (Ang, 2023; Zeilinger et al., 2023). Exclusion criteria were as follows: (1) preoperative corrected distant visual acuity (CDVA) < 0.3 LogMAR; (2) history of corneal refractive surgery (Vrijman et al., 2019; Wang and Koch, 2021; Cione et al., 2023) or corneal transplantation; (3) history of ocular diseases, including keratoconus, glaucoma, severe ocular surface diseases, retinal diseases and history of retina or vitrectomy; (4) corneal endothelium density less than 2000 cells/mm^2^; (5) dilated pupil diameter < 6 mm; (6) poor optical coherence tomography image of macula (Sample picture was placed in Supplementary Figure S1) or previous macular disease; (7) not enough valid data obtained from IOL master 700; (8) vestibular dysfunction and extraocular muscle abnormalities that affect the free movement of eyes; (9) cognitive disorders or other systemic diseases causing poor cooperation.

### 2.2 Randomization and masking

A total of 169 patients were assessed for eligibility, and 124 patients were enrolled monocularly for cataract surgery. For patients who met inclusion criteria for both eyes, the eye with worse BCVA was included. The enrolled patients were randomly assigned 1: 1 to receive toric trifocal (AT LISA toric 939 M/MP, Carl Zeiss Meditec AG, Jena, Germany) or toric bifocal (AT LISA toric 909 M Carl Zeiss Meditec AG, Jena, Germany) IOL implantation. Allocation was determined by investigators with a web-based central randomization system.^1^ Patients and investigators were masked to the allocation during the research.

### 2.3 Preoperative and postoperative evaluation

All recruited patients underwent a detailed preoperative evaluation, including uncorrected and corrected distance (4 m, UDVA/CDVA), intermediate (80 cm, UIVA/DCIVA) and near (40 cm for trifocal and 35 cm for bifocal IOL group, UNVA/DCNVA) visual acuity with LogMAR visual chart, monocular uncorrected and corrected distance DVA (UDDVA/CDDVA) with a self-developed program, noncontact tonometer (NT-530P, NIDEK Co., Ltd., Gamagori, Japan, v1.11), manifest refraction, slip-lamp biomicroscopy (BQ 900, Haag-Streit International, Köniz, Switzerland), IOL master 700 (Carl Zeiss Meditec AG, Jena, Germany, v1.90.38.02), corneal topography (Pentacam, Oculus Optikgeräte GmbH, Wetzlar, Germany, v1.21r43), and non-mydriatic fundus photography (TRC-NW400, Topcon Corporation, Tokyo, Japan, v1.1). IOL diopter and cylindrical power were calculated with the Z CALC Online IOL Calculator^2^ targeting Plano. Surgically induced astigmatism was set as 0.5D and A-constants were 118.4 and 118.5 for toric bifocal and trifocal IOL, respectively.

All patients were scheduled to be examined one day, one week, one month and three months postoperatively. One day postoperatively, monocular UDVA, CDVA, UIVA, DCIVA, UNVA, DCNVA, noncontact intraocular pressure and slip-lamp biomicroscopy were assessed. Monocular manifest refraction and monocular uncorrected and corrected distance DVA (UDDVA and CDDVA) of 20, 40, and 80 degrees per second (dps) were further evaluated at one week, one month and three months postoperatively. The corrected DVA was examined after the residue refractive error fully corrected with spectacles.

### 2.4 Dynamic visual acuity testing procedure

The DVA test procedure was consistent with previous studies (Wang et al., 2022, 2023). The optotypes were presented on a 24-inch In-Plane Switching screen with a resolution of 1920 × 1,080 pixels (refresh rate of 60 Hz, brightness of 30 lux). The size and configuration of the moving optotype were designed according to the standard logarithmic visual chart, and the velocity was quantified with the viewing angle (degree) changes per second (dps). Optotype generation and presentation were controlled using Matlab2017b (MathWorks, United States).

Subjects were required to sit 3 meters away from the screen. They were required to identify the direction of optotypes which moved horizontally from left to right in the middle of the screen. If more than 5 out of 8 optotypes were accurately identified, the size of the optotype would be reduced until the minimum optotype was attained. The result was calculated using following equation:


$$DVA=-\log_{10}A-\frac{0.1}{8}*B$$

A: the minimum size of optotype; B: the number of optotype that could be recognized in smaller size.

### 2.5 Surgical procedures

Cataract surgery was performed by the same surgeon under topical anesthesia. Prior to surgery, eligible images from IOL Master 700 were obtained to perform digital guidance for IOL alignment. Followed by an injection of viscoelastic in an assisted incision at 3 o’clock, a 3.2 mm clear corneal incision at 11 o’clock position was performed. Next, continuous circular capsulorhexis of 5.0 to 5.5 mm and hydrodissection were performed. After phacoemulsification (Centurion vision system, Alcon laboratories, Inc., Texas, United States), irrigation-aspiration to clear the residue cortex, toric trifocal or bifocal IOL was implanted through the main incision into the capsule under the navigation of CALLISTO eye makeless alignment (Carl Zeiss Meditec AG, Jena, Germany). After the surgery, all patients were given 0.5% Levofloxacin drops (Santen Pharmaceutical Co., Ltd., Osaka, Japan) and 1% Prednisolone Acetate Ophthalmic Suspension (Pred Forte, Allergan Inc., Mayo, Ireland) four times per day for one month. The dosage of preservative-free artificial tears was adjusted according to patients’ symptoms.

### 2.6 Statistical analysis

To calculate the required number of study subjects, we conducted a power analysis with PASS 15 using the data from a previous study that evaluated DVA in patients implanted with monofocal and trifocal IOL (Ren et al., 2020). A sample size of 118 subjects (59 per group) could achieve 90% power at a 0.05 significance level with factoring in an attrition rate of 20% (Ren et al., 2020).

Statistical analysis was performed using IBM SPSS 26.0 (IBM, Armonk, New York, United States). Continuous variables were shown in mean and standard deviation, and categorical variables were presented with the number (percentage). The Kolmogorov–Smirnov test was applied to test normal distribution. Chi-square test was applied for categorical variables. An Independent sample *t*-test was applied to compare normally distributed continuous variables between two groups, and for the continuous variables with non-normal distribution, the nonparametric test was applied. Generalized estimating equations were applied to compare the postoperative DVA at different follow-up time points and different speeds, considering the main effect of IOL type, static visual acuity and age. Spearman or Pearson’s correlation was conducted to analyze the relationship between DVA three months after surgery and age, postoperative static vision and refraction according to normality of data. We further performed multiple linear regression to analyze potential influential factors for DVA at three months. Age, SE, and UDVA/CDVA were included as potential factors. Bonferroni correction was conducted for multiple comparisons. *p* < 0.05 was considered statistically significant.

## 3 Results

### 3.1 Baseline clinical features

**Figure 1:**
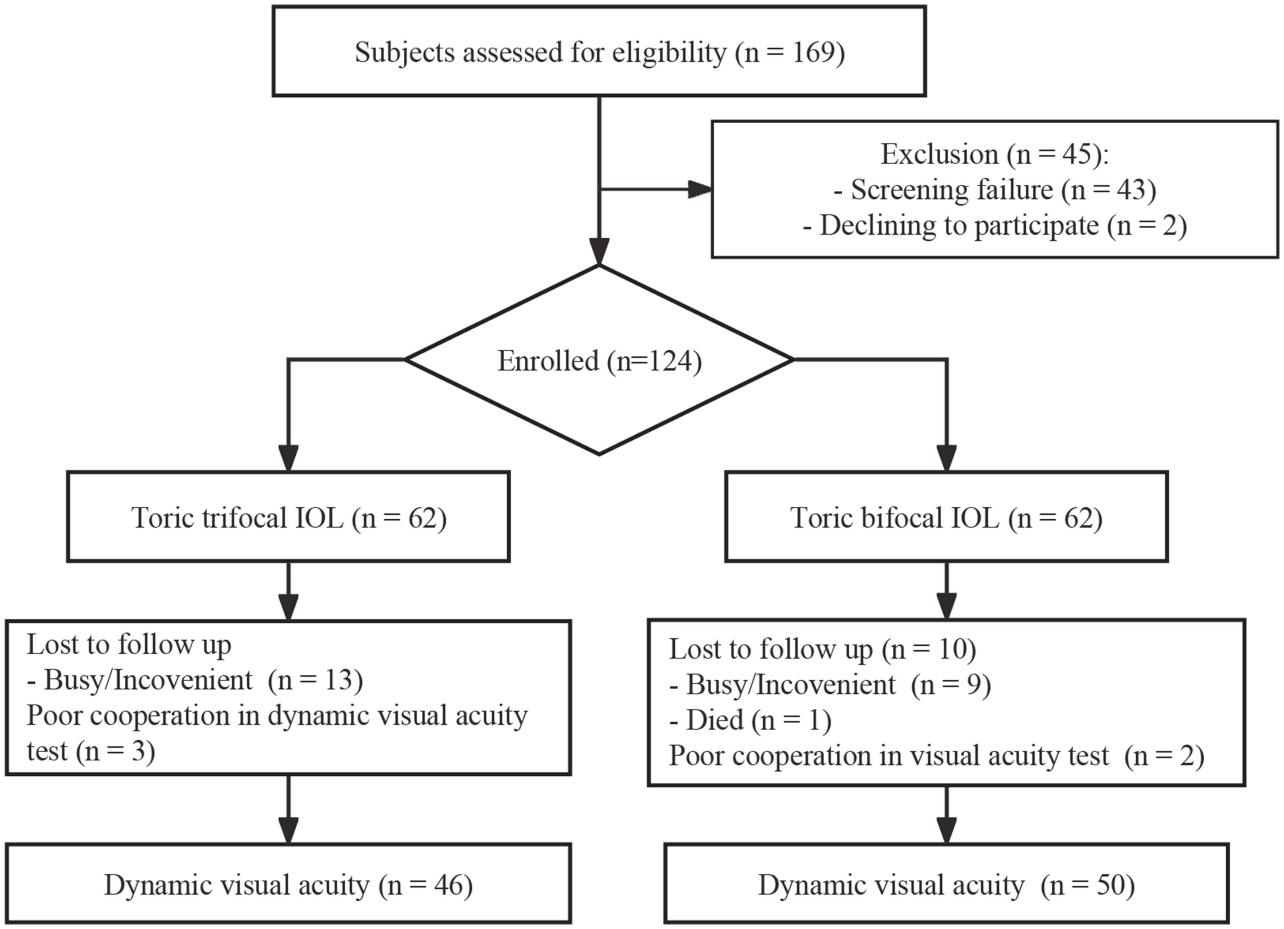
A flowchart showing participants enrolled.

A total of 169 patients were assessed for eligibility, and 124 eyes of 124 patients were recruited into the study, 62 eyes in each group. Twenty-three of them were excluded for loss to follow up. Two patients were excluded due to poor cooperation during visual acuity test and three were excluded from DVA analysis due to poor cooperation during DVA test. Among the 28 withdrawal patients, 16 received trifocal IOL, and the other 12 patients received bifocal IOL implantation (Figure 1). Ultimately, a total of 99 eyes of 99 patients with a mean age of 67.71 ± 10.29 years were included in the static vision analysis. And 96 eyes of 96 patients were included in the dynamic vision analysis with mean age of 67.53 ± 10.23 years, and males accounted for 45.8%. The baseline clinical characteristics are summarized in Table 1. There were no significant differences in age, sex, AL, corneal astigmatism, UDVA, CDVA, sphere, cylinder, SE and CDDVA at 20, 40 and 80 dps between the two groups (*p* > 0.05, respectively).

**Table 1:** Baseline clinical characteristics.

	Toric trifocal IOL group	Toric bifocal IOL group	*p* value
Subjects (n)^a^	49	50	-
Sex (male, %)	24 (49.0%)	21 (42%)	0.486
Age (yrs)	68.96 ± 11.72	66.48 ± 8.61	0.233
Axis length (mm)	24.22 ± 1.54	23.78 ± 1.41	0.142
Corneal astigmatism (D)^b^	1.30 ± 0.74	1.29 ± 0.5	0.943
LogMAR UDVA	0.60 ± 0.27	0.59 ± 0.31	0.887
LogMAR CDVA	0.37 ± 0.21	0.38 ± 0.25	0.767
Sphere (D)	−1.62 ± 3.94	−1.52 ± 3.99	0.901
Cylinder (D)	−1.51 ± 1.37	−1.38 ± 1.08	0.532
Spherical equivalent (D)	−2.37 ± 4.05	−2.21 ± 3.99	0.837
CDDVA at 20 dps	0.5902 ± 0.2041	0.5841 ± 0.2383	0.906
CDDVA at 40 dps	0.8122 ± 0.2600	0.8216 ± 0.2753	0.880
CDDVA at 80 dps	0.9419 ± 0.2907	0.9216 ± 0.3116	0.773

^a^Data were obtained from 99 patients underwent 3 months follow-up. ^b^Corneal astigmatism: ΔTK in IOL master 700. CDVA, corrected visual acuity; DCIVA, distant-corrected intermediate visual acuity; DCNVA, distant-corrected near visual acuity; CDDVA at 20/40/80 dps, corrected dynamic distance visual acuity at 20, 40, and 80 degree per second; D, diopter; UDVA, uncorrected distance visual acuity; UIVA, uncorrected intermediate visual acuity; UNVA, uncorrected near visual acuity.

### 3.2 Static vision and refraction

The static vision outcome and refraction at three months are summarized in Table 2. Significant better UIVA (trifocal, 0.17 ± 0.13 vs. bifocal, 0.23 ± 0.13, *p* = 0.037) and DCIVA (trifocal, 0.16 ± 0.11 vs. bifocal, 0.20 ± 0.12, *p* = 0.048) were observed in patients implanting with toric trifocal than bifocal IOL at three months postoperatively. Nevertheless, there was no significant difference in UDVA, CDVA, UNVA and DCNVA between two groups. The mean postoperative SE was 0.19 ± 0.40 D for the toric trifocal group, which was closer to the Plano target than the toric bifocal group (0.50 ± 0.46 D, *p* < 0.001).

**Table 2:** Postoperative static visual acuity and refraction at 3 months.

	Toric trifocal IOL group	Toric bifocal IOL group	*P* value
Subjects (n)^a^	49	50	*-*
UDVA	0.13 ± 0.11	0.14 ± 0.13	0.683
UIVA	0.17 ± 0.13	0.23 ± 0.13	0.037^*^
UNVA	0.24 ± 0.11	0.26 ± 0.15	0.522
CDVA	0.09 ± 0.12	0.06 ± 0.1	0.122
DCIVA^b^	0.16 ± 0.11	0.20 ± 0.12	0.048^*^
DCNVA^b^	0.22 ± 0.11	0.21 ± 0.14	0.581
Sphere (D)	0.19 ± 0.40	0.50 ± 0.46	0.001^*^
Cylinder (D)	−0.43 ± 0.47	−0.30 ± 0.4	0.131
SE (D)	−0.02 ± 0.40	0.35 ± 0.44	<0.001^*^
ABS-SE (D)	0.28 ± 0.28	0.44 ± 0.35	0.040^*^

CDVA, corrected visual acuity; DCIVA, distant-corrected intermediate visual acuity; DCNVA, distant-corrected near visual acuity; D, diopter; SE, Spherical equivalent; ABS-SE, absolute value of spherical equivalent; UDVA, uncorrected distance visual acuity; UIVA, uncorrected intermediate visual acuity; UNVA, uncorrected near visual acuity. *Statistically significant. ^a^Data were obtained from 99 patients underwent 3 months follow-up. ^b^DCIVA and DCNVA were impervious to SE.

### 3.3 Dynamic visual acuity outcome

**Figure 2:**
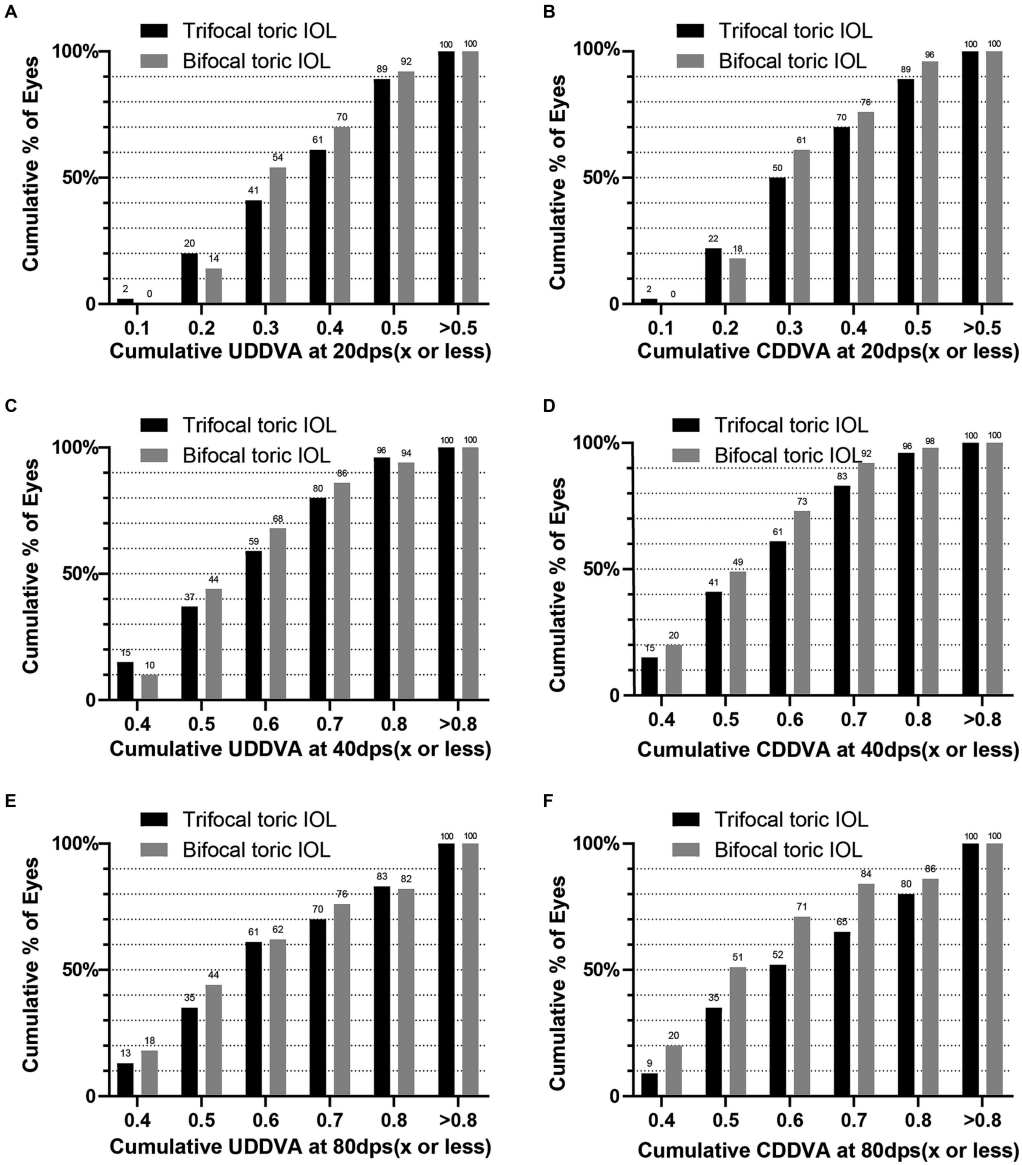
Cumulative postoperative uncorrected and corrected dynamic distance visual acuity at 20, 40 and 80dps: **(A)** cumulative UDDVA at 20 dps; **(B)** cumulative CDDVA at 20 dps; **(C)** cumulative UDDVA at 40 dps; **(D)** cumulative CDDVA at 40 dps; **(E)** cumulative UDDVA at 80 dps; **(F)** cumulative CDDVA at 80 dps.

The histogram of cumulative UDDVA and CDDVA at three months postoperatively is demonstrated in Figure 2. The DVA at each follow-up time point in the two groups is demonstrated in Table 3. At three months postoperatively, the mean CDDVA at 80 dps was 0.5607 ± 0.2032 for the trifocal group, which was significantly better than patients implanting with bifocal IOL (0.6573 ± 0.2450, *p* = 0.039). No significant difference was revealed in UDDVA or CDDVA at 20 and 40 dps between the two groups (*p* > 0.05, respectively). With increasing speed, the CDDVA (*p* < 0.001) and UDDVA (*p* < 0.001) decreased in both toric trifocal and bifocal IOL groups. No significant changes were found among different time points in UDDVA at 20, 40 and 80 dps and CDDVA at 20 dps (*p* > 0.05, respectively). There was a significant improvement in 40 dps CDDVA at one month (*p* = 0.032) and three months (*p* = 0.008) than that at one week postoperatively.

**Table 3:** Postoperative dynamic visual acuity at 3 months.

	Time points	Toric trifocal IOL group	Toric bifocal IOL group	*P* value
Subjects (n)^a^		46	50	-
UDDVA (20 dps)	One week	0.3982 ± 0.1885	0.3426 ± 0.1606	0.151
	One month	0.3540 ± 0.1475	0.3434 ± 0.1392	0.722
	Three months	0.3533 ± 0.1467	0.3383 ± 0.1296	0.596
UDDVA (40 dps)	One week	0.6463 ± 0.2337	0.5863 ± 0.2072	0.219
	One month	0.5864 ± 0.1953	0.5533 ± 0.1865	0.403
	Three months	0.5821 ± 0.1761	0.5628 ± 0.1488	0.562
UDDVA (80 dps)	One week	0.7378 ± 0.3018	0.6503 ± 0.2434	0.151
	One month	0.6556 ± 0.2431	0.6508 ± 0.2301	0.922
	Three months	0.6313 ± 0.2444	0.6048 ± 0.2313	0.587
CDDVA (20 dps)	One week	0.3771 ± 0.1725	0.3333 ± 0.1922	0.369
	One month	0.3364 ± 0.1494	0.3353 ± 0.1419	0.972
	Three months	0.3332 ± 0.1507	0.3145 ± 0.1196	0.505
CDDVA (40 dps)	One week	0.6196 ± 0.2196	0.5986 ± 0.2255	0.724
	One month	0.5729 ± 0.1922	0.5561 ± 0.1822	0.701
	Three months	0.5707 ± 0.1895	0.5263 ± 0.1412	0.197
CDDVA (80 dps)	One week	0.7167 ± 0.2999	0.6667 ± 0.2546	0.503
	One month	0.6361 ± 0.2544	0.6192 ± 0.2290	0.765
	Three months	0.6573 ± 0.2450	0.5607 ± 0.2032	0.039^*^

CDDVA at 20/40/80 dps, corrected dynamic distance visual acuity at 20, 40, and 80 degree per second; UDDVA at 20/40/80 dps, uncorrected dynamic distance visual acuity at 20, 40, and 80 degree per second. ^*^Statistically significant. ^a^Data were obtained from 96 patients underwent dynamic visual acuity evaluation at 3 months.

### 3.4 The influential factor analysis for postoperative DVA

The results of Spearman’s correlation analysis between DVA and associated parameters at three months postoperatively are summarized in Table 4. Postoperative UDDVA at 20, 40 and 80 dps were significantly positively correlated with UDVA (*p* < 0.05, respectively), UIVA (*p* < 0.05, respectively) and UNVA (*p* < 0.05, respectively) and postoperative CDDVA at 20, 40 and 80 dps were positively associated with CDVA (*p* < 0.05, respectively), DCIVA (*p* < 0.05, respectively) and DCNVA (*p* < 0.05, respectively) at three months. A significant positive correlation was observed between age and postoperative UDDVA and CDDVA at 20, 40 and 80 dps (*p* < 0.05 for all the analyses). Positive correlations were found between postoperative SE and UDDVA at 80 dps in toric bifocal IOL group (*R* = 0.334, *p* = 0.018) and all patients with Spearman’s correlation analysis (*R* = 0.250, *p* = 0.014).

**Table 4:** Spearman correlation between DVA at 3 months and potential factors.

			Age	SE (D)	UDVA	UIVA	UNVA
Toric trifocal IOL group	UDDVA (20 dps)	R	0.541^*^	0.183	0.707^*^	0.559^*^	0.610^*^
		*P*	<0.001	0.225	<0.001	<0.001	<0.001
	UDDVA (40 dps)	R	0.465^*^	0.037	0.686^*^	0.475^*^	0.596^*^
		*P*	0.001	0.808	<0.001	0.001	<0.001
	UDDVA (80 dps)	R	0.406^*^	0.256	0.661^*^	0.410^*^	0.497^*^
		*P*	0.005	0.085	<0.001	0.005	<0.001
			Age	SE (D)	CDVA	DCIVA	DCNVA
	CDDVA (20 dps)	R	0.497^*^	0.186	0.728^*^	0.558^*^	0.600^*^
		*P*	<0.001	0.215	<0.001	<0.001	<0.001
	CDDVA (40 dps)	R	0.445^*^	0.043	0.654^*^	0.458^*^	0.517^*^
		*P*	0.002	0.777	<0.001	0.001	<0.001
	CDDVA (80 dps)	R	0.403^*^	0.227	0.648^*^	0.480^*^	0.507^*^
		*P*	0.005	0.129	<0.001	0.001	<0.001
Toric bifocal IOL group			Age	SE (D)	UDVA	UIVA	UNVA
	UDDVA (20 dps)	R	0.429^*^	0.145	0.473^*^	0.547^*^	0.482^*^
		*P*	0.002	0.314	0.001	<0.001	<0.001
	UDDVA (40 dps)	R	0.369^*^	0.224	0.430^*^	0.442^*^	0.410^*^
		*P*	0.008	0.118	0.002	0.001	0.003
	UDDVA (80 dps)	R	0.394^*^	0.334^*^	0.535^*^	0.524^*^	0.566^*^
		*P*	0.005	0.018	<0.001	<0.001	<0.001
			Age	SE (D)	CDVA	DCIVA	DCNVA
	CDDVA (20 dps)	R	0.350^*^	0.006	0.405^*^	0.491^*^	0.310^*^
		*P*	0.014	0.969	0.004	<0.001	0.03
	CDDVA (40 dps)	R	0.303^*^	−0.011	0.459^*^	0.439^*^	0.378^*^
		*P*	0.035	0.942	0.001	0.002	0.007
	CDDVA (80 dps)	R	0.331^*^	0.175	0.322^*^	0.363^*^	0.296^*^
		*P*	0.02	0.23	0.024	0.01	0.039
Total			Age	SE (D)	UDVA	UIVA	UNVA
	UDDVA (20 dps)	R	0.495^*^	0.128	0.581^*^	0.527^*^	0.527^*^
		*P*	<0.001	0.215	<0.001	<0.001	<0.001
	UDDVA (40 dps)	R	0.429^*^	0.094	0.550^*^	0.433^*^	0.482^*^
		*P*	<0.001	0.362	<0.001	<0.001	<0.001
	UDDVA (80 dps)	R	0.401^*^	0.250^*^	0.590^*^	0.444^*^	0.525^*^
		*P*	<0.001	0.014	<0.001	<0.001	<0.001
			Age	SE (D)	CDVA	DCIVA	DCNVA
	CDDVA (20 dps)	R	0.444^*^	0.063	0.593^*^	0.497^*^	0.440^*^
		*P*	<0.001	0.543	<0.001	<0.001	<0.001
	CDDVA (40 dps)	R	0.400^*^	−0.037	0.581^*^	0.405^*^	0.435^*^
		*P*	<0.001	0.724	<0.001	<0.001	<0.001
	CDDVA (80 dps)	R	0.385^*^	0.095	0.520^*^	0.362^*^	0.389^*^
		*P*	<0.001	0.362	<0.001	<0.001	<0.001

CDVA, corrected visual acuity; CIVA, distant-corrected corrected intermediate visual acuity; CNVA, distant-corrected near visual acuity; CDDVA at 20/40/80 dps, corrected dynamic distance visual acuity at 20, 40, and 80 degree per second; SE, Spherical equivalent; UDVA, uncorrected distance visual acuity; UIVA, uncorrected intermediate visual acuity; UNVA, uncorrected near visual acuity; UDDVA at 20/40/80 dps, uncorrected dynamic distance visual acuity at 20, 40, and 80 degree per second. *Statistically significant.

Multiple linear regression was performed to investigate the influential factors for postoperative DVA at three months, and the results are demonstrated in Table 5. The postoperative UDDVA at 20, 40 and 80 dps were significantly associated with age (20 dps, *p* < 0.001, 40 dps, *p* = 0.001, 80 dps, *p* = 0.001) and UDVA (20 dps, *p* < 0.001, 40 dps, *p* < 0.001, 80 dps, *p* < 0.001). Similarly, the postoperative CDDVA at 20, 40 and 80 dps were significantly correlated with age (20 dps, *p* = 0.001, 40 dps, *p* = 0.004, 80 dps, *p* = 0.024) and CDVA (20 dps, *p* < 0.001, 40 dps, *p* < 0.001, 80 dps, *p* < 0.001). In addition, CDDVA at 80 dps was significantly correlated with IOL group (*p* = 0.045).

**Table 5:** Multiple linear regression for DVA at 3 months.

	Dependent variable	Independent variable	Standardized coefficient	*P*
Toric trifocal IOL group	UDDVA (20 dps)	Age	0.392	<0.001^*^
		UDVA	0.615	<0.001^*^
	UDDVA (40 dps)	Age	0.347	0.002^*^
		UDVA	0.636	<0.001^*^
	UDDVA (80 dps)	Age	0.234	0.05
		UDVA	0.584	<0.001^*^
	CDDVA (20 dps)	Age	0.332	0.002^*^
		CDVA	0.643	<0.001^*^
	CDDVA (40 dps)	Age	0.328	0.006^*^
		CDVA	0.591	<0.001^*^
	CDDVA (80 dps)	Age	0.236	0.052
		CDVA	0.576	<0.001^*^
Toric bifocal IOL group	UDDVA (20 dps)	Age	0.302	0.027^*^
		UDVA	0.369	0.008^*^
	UDDVA (40 dps)	UDVA	0.326	0.022^*^
	UDDVA (80 dps)	UDVA	0.42	0.002^*^
	CDDVA (20 dps)	CDVA	0.323	0.029^*^
	CDDVA (40 dps)	CDVA	0.404	0.007^*^
	CDDVA (80 dps)	CDVA	0.275	0.067
Total	UDDVA (20 dps)	Age	0.357	<0.001^*^
		UDVA	0.484	<0.001^*^
		IOL group	−0.023	0.787
	UDDVA (40 dps)	Age	0.296	0.001^*^
		UDVA	0.474	<0.001^*^
		IOL group	−0.027	0.772
	UDDVA (80 dps)	Age	0.220	0.001^*^
		UDVA	0.498	<0.001^*^
		IOL group	−0.110	0.217
	CDDVA (20 dps)	Age	0.288	0.001^*^
		CDVA	0.511	<0.001^*^
		IOL group	0.022	0.805
	CDDVA (40 dps)	Age	0.258	0.004^*^
		CDVA	0.499	<0.001^*^
		IOL group	−0.015	0.867
	CDDVA (80 dps)	Age	0.209	0.024^*^
		CDVA	0.437	<0.001^*^
		IOL group	0.042	0.045^*^

CDVA, corrected visual acuity; CDDVA at 20/40/80 dps, corrected dynamic distance visual acuity at 20, 40, and 80 degree per second; SE, Spherical equivalent; UDVA, uncorrected distance visual acuity; UDDVA at 20/40/80 dps, uncorrected dynamic distance visual acuity at 20, 40 and 80 degree per second. ^*^Statistically significant.

## 4 Discussion

The present study compared DVA in age-related cataract patients implanted with toric trifocal or bifocal IOL and explored potential influence factors of postoperative DVA. We found that the toric trifocal IOL group obtained better uncorrected and corrected intermediate static visual acuity than the toric bifocal IOL group, but bifocal toric IOL implantation provided better dynamic visual acuity at high speed. Meanwhile, age and postoperative static vision were the main influential factors of postoperative dynamic visual acuity.

Both AT LISA toric 939 M/MP and AT LISA toric 909 M are single-piece IOL made of hydrophilic acrylate with a water content of 25%, possessing a hydrophobic surface and a diffractive structure dividing incoming light to provide a range of vision from distant to near space within optic diameter of 6 mm. The add powers within the 4.34 mm diameter are +3.33 D for the near focal point and + 1.66 D for the intermediate focal point at the IOL plane, and the add power between 4.34 mm and 6 mm diameter area is +3.75 D for AT LISA toric 939 M/MP. According to the manufacturer, the IOL allocates approximately 50% of light to far, 20% to intermediate, and 30% to near for a 4.5 mm pupil, and more light energy will be allocated to near with larger pupil. AT LISA toric 909 M has +3.75 D near add at the IOL plane and the incident light is distributed with 65% to distance focus and 35% to near focus (Mojzis, 2019; Piovella et al., 2019).

Significant differences in UIVA (0.17 ± 0.13 vs. 0.23 ± 0.13) and DCIVA (0.16 ± 0.11 vs. 0.20 ± 0.12, respectively) were found in favor of trifocal group at 3 months follow-up which was in accord with the design of the two IOLs. Previous studies also obtained similar result that toric trifocal group presented better UIVA and DCIVA than the toric bifocal group (Bozkurt Gencer et al., 2021; Tekce and Gulmez, 2021). It is worth mentioning that significant differences were observed both in SE and absolute value of SE, and bifocal group presented higher degree of hypermetropia. UIVA might be affected by refractive error, and with SE closer to farsightedness, bifocal IOL group tended to present worse UIVA. Since significant better CDIVA was obtained in trifocal IOL group which was impervious to SE, the difference in intermediate visual acuity between the two group was clinically relevant.

DVA was evaluated with a self-developed test system which has been applied in patients who underwent corneal refractive surgery and cataract surgery (Ao et al., 2014; Ren et al., 2020; Wang et al., 2023). In the present study, CDDVA at 80 dps in the toric bifocal group was significantly better than that in the trifocal group, but the CDVA was comparable between the two groups. According to Huygens-Fresnel principle, by modulating the microstructures in the concentric zone of intraocular lens, the zero, first and second order of diffraction were utilized to create different focuses and the incident light was allocated to different focuses (Alió and Pikkel, 2019). 50 and 65% incident light were allocated to distance focus in the toric trifocal and bifocal IOL, respectively. It is believed that as light intensity falls, the photon shot noise relatively increases (Stöckl et al., 2017). When same amount of light entering eyes, more lights were allocated to the distance focal point in patients implanted with bifocal IOLs (Vega et al., 2014; Mojzis, 2019), reduced visual function may obtained in trifocal IOL group with lower signal and relatively higher noise. Thus, it could be speculated that when observing objects, more light power from the optotype could allocate to distance focus, and might provide better dynamic visual acuity in bifocal IOL group. Different from the outcome of high-speed DVA, the CDDVA at 20 and 40 dps were not significantly different between the two groups. According to previous research, for the object with a speed of less than 50 dps, the observer could apply smooth pursuit to catch up with the moving object (France, 1994) and static visual acuity was only related to dynamic-object DVA at slow speed (Palidis et al., 2017). Thus, static vision acuity is more closely associated with low-speed DVA than high-speed DVA in the present study. The comparable low-speed DVA between the two groups might be due to similar static distance visual acuity between the two groups.

The influential factor analysis demonstrated that postoperative DVA worsened with increasing age. Previous research showed that DVA increased during childhood, peaked during the 20s, and gradually decreased during middle and old age (Ishigaki and Miyao, 1994; Wist et al., 2000; Lee et al., 2022). In the present research, the mean age of enrolled cataract patients was more than 60 years old. Thus, the result was largely consistent with the previous research. Eye movement is crucial for dynamic visual acuity. Compared with younger adults, older patients might have declined oculomotor abilities and peripheral awareness, which could be attributed to the worse DVA (Sharpe and Sylvester, 1978; Ren et al., 2020). We also showed that postoperative patients with better static vision tend to have better DVA. Static visual acuity influences the clearness of images on the central retina that affects the identification of moving objects. Thus, postoperative static visual acuity significantly correlated with postoperative DVA.

Certain limitations exist in the present study. Firstly, the follow-up was only extended to three months postoperatively. Larger samples and longer-term studies are needed in the future. We evaluated DVA with horizontal moving optotypes in the present research. DVA with other moving patterns should be further evaluated. The advantage of multifocal IOL is to provide outstanding intermediate and near vision for patients. Thus, intermediate and near DVA should be further investigated to provide information on full-range DVA in post-IOL implantation patients. Moreover, we only compared toric trifocal and bifocal IOLs, a control group of toric monofocal IOL should be included in the future. And in cases of bilateral eyes, eyes with worse BCVA were included in the present research, which reduced the extrapolability to patients with better visual acuity. Lastly, the testing distance was 4 m when evaluating static visual acuity which may impact the accuracy of distance visual acuity, a 6-m distance testing is need to be utilized in our future work (Hoffer and Savini, 2021).

In summary, from the three-month observation, age-related cataract patients implanted with toric trifocal IOL obtained better uncorrected and corrected intermediate static visual acuity than patients implanted with toric bifocal IOL. Toric bifocal IOL implantation provided better distance dynamic visual acuity at high speed than trifocal implantation. Age and postoperative static vision were the main influential factors for postoperative dynamic visual acuity. The present study provides the basis for applying DVA as an effective and sensitive indicator for functional vision evaluation in cataract patients after IOL implantation. With further improvement, DVA could guide functional IOL selection in patients with different postoperative requirements.

## Data availability statement

The original contributions presented in the study are included in the article/Supplementary material, further inquiries can be directed to the corresponding author.

## Ethics statement

The studies involving humans were approved by Peking University Third Hospital Medical Science Research Ethics Committee. The studies were conducted in accordance with the local legislation and institutional requirements. The participants provided their written informed consent to participate in this study.

## Author contributions

YL: Formal analysis, Investigation, Methodology, Visualization, Writing – original draft. XiL: Conceptualization, Methodology, Project administration, Visualization, Writing – review & editing. XJ: Conceptualization, Investigation, Methodology, Writing – review & editing. YW: Conceptualization, Investigation, Methodology, Software, Writing – review & editing. TW: Formal analysis, Investigation, Software, Writing – review & editing. HX: Investigation, Software, Writing – review & editing, Visualization. XuL: Conceptualization, Funding acquisition, Methodology, Project administration, Resources, Supervision, Writing – review & editing.

## Footnotes

^1^ https://edc.clinflash.net

^2^ https://zcalc.meditec.zeiss.com
