# Supplementary figures and images for: Comparison of dynamic visual acuity after implantation of toric bifocal or trifocal intraocular lens in age-related cataract patients: a randomized controlled trial
Source: Front Neurosci. 2023 Dec 21;17:1287626. doi: 10.3389/fnins.2023.1287626 (PMC10765614; doi:10.3389/fnins.2023.1287626)

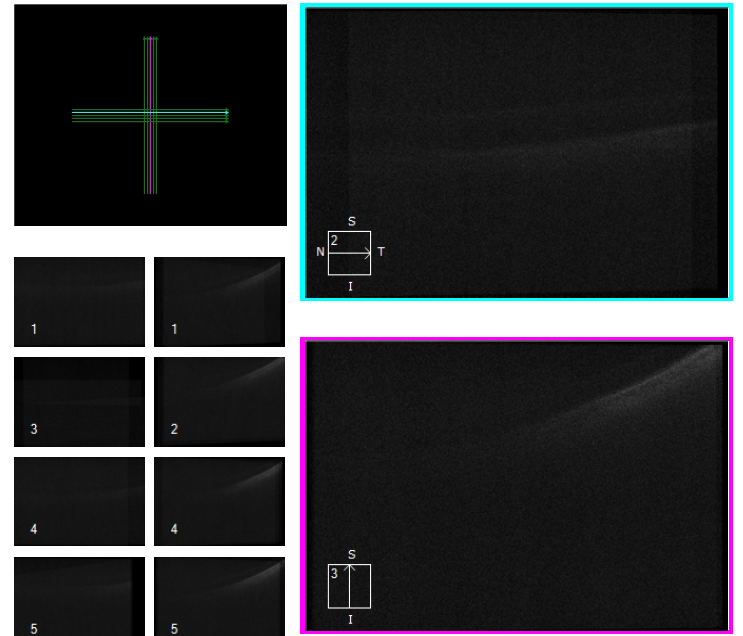

Supplement: Supplementary file 1 [file Image_1.TIF]
